# Commentary: Parkinson's Disease Genes VPS35 and EIF4G1 Interact Genetically and Converge on α-Synuclein

**DOI:** 10.3389/fnins.2016.00162

**Published:** 2016-04-18

**Authors:** Diana A. Olszewska, Allan McCarthy, Tim Lynch

**Affiliations:** ^1^Department of Neurology, Dublin Neurological Institute at the Mater Misericordiae HospitalDublin, Ireland; ^2^The Adelaide and Meath HospitalDublin, Ireland

**Keywords:** *VPS35*, Parkinson's disease, *EIF4G1*

Several loci and genes are associated with Parkinson's disease (IPD) with some interacting at a cellular level. For example PINK1 activates Parkin through phosphorylation of ubiquitin and enhances Parkin-mediated elimination of damaged mitochondria (mitophagy). Mutations in both may result in deregulation of mitochondrial homeostasis leading to neurodegeneration (Olszewska et al., 2014). Intimate knowledge of the interplay between gene products will be crucial for targeted-therapeutic development and pathway-based treatment initiatives (e.g., Parkin activators to enhance its housekeeping abilities in PD; Kazlauskaite et al., 2014).

Dhungel et al. (2015) reported an unexpected interaction between two rare IPD genes: *VPS35* (Vacuolar sorting protein 35) and *EIF4G1* (Eukaryotic translation initiation factor 4 gamma 1). It is important to understand the role of *VPS35* and *EIF4G1* first to fully appreciate complex interactions between them.

*VPS35* is a subunit of the retromer complex first described in yeast in 1998 (Small and Petsko, 2015). The retromer is crucial in mediating retrograde transport of specific membrane proteins (cargo; Seaman, 2012) from endosomes back to the trans-Golgi network (hydrolases and proteases) and to the cell surface (Small and Petsko, 2015). In yeast the retromer consists of 5 proteins encoded by vacuole sorting proteins (VPS) genes: the cargo recognition trimer (VPS 26, 29, and 35) and trimer recruitment dimer (VPS 5 and 17). Eukaryote proteins corresponding to VPS5 and VPS17 are members of the sorting nexin (Snx) family called Snx-BAR proteins (Seaman, 2012) and mediate tubule or vesicle formation into which cargo is assigned for different cellular destinations. The retromer has an important role in secretion of signaling proteins (Wnt) and apoptotic cell clearance. Absence of retromer activity causes decreased Wnt secretion and dopaminergic neuronal loss (Seaman, 2012). Moreover the retromer transports VPS10, an important receptor family, is linked to Alzheimer's disease and frontotemporal dementia (Lane et al., 2012).

*VPS35* is a central scaffold for binding of other subunits of the retromer. Decreased levels of *VPS35* and VPS26 were shown in brain regions susceptible to Alzheimer's disease in mice (Muhammad et al., 2008). Mutations in *VPS35* (p.Asp620Asn) are associated with familial and rarely sporadic young-onset levodopa-responsive IPD without dementia providing evidence of the retromer involvement in neurodegeneration (Bonifati, 2014). The mutation frequency is reported at 1% in Caucasians and Japanese (Sudhaman et al., 2013).

*EIF4G1* encodes a protein which has a role in protein regulation. EIF4G1 is part of a multi-subunit translation initiation complex regulating recruitment of mRNA to the ribosome. The pathogenicity of *EIF4G1* mutations is debated. A R1205H variant (Chartier-Harlin et al., 2011) was reported in a family with 10 affected members and mild course of levodopa-responsive disease and one sporadic Irish patient (McCarthy, 2014). A502V variant was also found in two other PD patients. The R12O5H co-segregated with the disease and both R1205H, A502V mutations were absent in controls. In addition the binding within the translation initiation complex and formation of the larger complex were impaired indicating loss-of-function in keeping with a neurodegenerative disorder and was supportive of the pathogenicity of *EIF4G1* mutations (Chartier-Harlin et al., 2011).

Subsequently evidence has suggested that R1205H is only a benign polymorphism (Dhungel et al., 2015) as a number of studies identified R1205H in both patients and controls (Nuytemans et al., 2013; Dhungel et al., 2015; Nichols et al., 2015). The majority of South African and Asian studies (Quadri et al., 2013; Nishioka et al., 2014; Weng et al., 2015) failed to identify *EIF4G1* mutations either in patients or in controls (Sudhaman et al., 2013). In Caucasians the mutations occur in 11.57% familial cases and 0.09% sporadic cases (Sudhaman et al., 2013).

The largest study (2146 sporadic and familial patients) examining the pathogenicity of *EIF4G1* mutations was reported in 2014 in Iceland (Huttenlocher et al., 2015). The R1205H was found in one sporadic PD patient and in 76 of 93,698 Icelanders over age 65 (five later found to have IPD symptoms). It was shown that *EIF4G1* is not a high-risk IPD locus and R1205H was not significantly associated with an increased IPD risk (Huttenlocher et al., 2015). The most recent meta-analysis demonstrated *EIF4G1* to be an extremely rare cause of IPD with the mutation frequency 0.23% for R1205H and 0.04% for A502V (Deng et al., 2015).

As both *VPS35* and *EIF4G1* are involved in protein signaling pathways, and interactions between them have been examined in multiple yeast and transgenic mice models in search for therapeutic options.

*EIF4G1* has two equivalents in yeast: TIF4631 and TIF4632. Dhungel at al. focused on the TIF4631 deletion which is closly related to humans and leads to global alteration in translation rate, unlike the TIF4632 deletion (Dhungel et al., 2015).

By the use of a Synthetic Genetic Array (SGA) 144 synthetic sick or lethal genes for TIF4631Δ (*EIF4G1* homolog) and 59 for VPS 35Δ were obtained (Dhungel et al., 2015). GO-Term-Finder was used to detect any important gene interactions. 14 lethal interactions were found to be common for both genes. A yeast ortholog of *ARFGAP1* encoding a GTPase –activating protein was common to both screens and has known interactions with, another IPD related gene, *LRRK2*. One of the strongest results from the TIF4631Δ screen was the equivalent of human Synaptojanin 1, recently connected to the early IPD (Quadri et al., 2013). The SGA is an efficient method which could potentially be used in the prediction of novel IPD genes.

Dhungel et al. did not find any lethal interaction between the yeast *EIF4G1* homolog and *VPS35*Δ (Dhungel et al., 2015). Upregulation of TIF4631, (in contrast to all other four yeast translation initiation factors) in *VPS35*Δ cells was highly toxic; however this effect was not seen in wild-type yeast cells. These results were replicated in human cells where *EIF4G1* upregulation caused translation-defects and accumulation of mis-folded proteins. While co-expression of *VPS35* was able to save the cells from toxicity, the expression of *VPS35* IPD-linked mutation (D620N) failed to provide protection (Dhungel et al., 2015).

Upregulation of *EIF4G1* (yeast TIF4631) was toxic in cells lacking VPS10. VPS10 acts downstream to *VPS35* and overexpression of VPS10 supresses the toxicity related to TIF4631 upregulation in *VPS35*Δ (Dhungel et al., 2015).

The authors examined the relation of both genes to alpha-synuclein accumulation by analyzing transgenic mice where upregulation of wild type *VPS35* rescued alpha-synuclein induced neurodegeneration, unlike upregulation of *VPS35* IPD-linked mutants (P316S and D620N) when increased hippocampal neuronal cell loss was found (Dhungel et al., 2015).

Dhungel et al. showed that the expression of *VPS35* suppresses alpha-synuclein accumulation and toxicity caused by *EIF4G1* upregulation. These proteins work in conjunction and the retromer and its components may be an important target for a drug discovery. Modulation of retromer function by increasing the level of *VPS35* could enhance intracellular transport. It was shown in pre-clinical studies that the enhancement of the retromer function reverses the neurotoxic effects without any obvious toxicity, however further studies are needed (Weng et al., 2015).

This study has suggested new therapeutic approaches in IPD involving modulation of *VPS35*, EIF4G, and retromer interactions. Although we are closer to understanding the molecular pathways underlying IPD, these results should be interpreted with caution in view of an on-going debate questioning *EIF4G1* pathogenicity and further studies to resolve this dilemma are needed.

## Author contributions

DO: writing the first draft, corrections. AM: review, corrections. TL: review, critique.

### Conflict of interest statement

The authors declare that the research was conducted in the absence of any commercial or financial relationships that could be construed as a potential conflict of interest.
